# Spatial Distribution of Focal Lesions in Whole-Body MRI and Influence of MRI Protocol on Staging in Patients with Smoldering Multiple Myeloma According to the New SLiM-CRAB-Criteria

**DOI:** 10.3390/cancers12092537

**Published:** 2020-09-07

**Authors:** Markus Wennmann, Thomas Hielscher, Laurent Kintzelé, Bjoern H. Menze, Georg Langs, Maximilian Merz, Sandra Sauer, Hans-Ulrich Kauczor, Heinz-Peter Schlemmer, Stefan Delorme, Hartmut Goldschmidt, Niels Weinhold, Jens Hillengass, Marc-André Weber

**Affiliations:** 1 Division of Radiology, German Cancer Research Center (DKFZ), Im Neuenheimer Feld 280, 69120 Heidelberg, Germany; h.schlemmer@dkfz.de (H.-P.S.); s.delorme@dkfz.de (S.D.); 2 Diagnostic and Interventional Radiology, University Hospital Heidelberg, Im Neuenheimer Feld 110, 69120 Heidelberg, Germany; laurent.kintzele@med.uni-heidelberg.de (L.K.); hans-ulrich.kauczor@med.uni-heidelberg.de (H.-U.K.); 3 Division of Biostatistics, German Cancer Research Center (DKFZ), Im Neuenheimer Feld 280, 69120 Heidelberg, Germany; t.hielscher@dkfz-heidelberg.de; 4 Department of Computer Science, Technical University of Munich, Boltzmannstrasse 3, 85748 Garching, Germany; bjoern.menze@tum.de; 5 Department of Biomedical Imaging and Image-Guided Therapy, Computational Imaging Research Laboratory, Medical University of Vienna, Währinger Gürtel 18-20, 1090 Vienna, Austria; georg.langs@meduniwien.ac.at; 6 Department of Medicine V, Multiple Myeloma Section, University Hospital Heidelberg, Im Neuenheimer Feld 410, 69120 Heidelberg, Germany; maximilian.merz@med.uni-heidelberg.de (M.M.); sandra.sauer@med.uni-heidelberg.de (S.S.); hartmut.goldschmidt@med.uni-heidelberg.de (H.G.); niels.weinhold@med.uni-heidelberg.de (N.W.); 7 National Center for Tumor Diseases (NCT), University Hospital Heidelberg, Im Neuenheimer Feld 410, 69120 Heidelberg, Germany; 8 Department of Medicine, Roswell Park Comprehensive Cancer Center, Elm and Carlton Streets, Buffalo, NY 14263, USA; jens.hillengass@roswellpark.org; 9 Institute of Diagnostic and Interventional Radiology, Paediatric Radiology and Neuroradiology, University Medical Centre Rostock, Ernst-Heydemann-Str. 6, 18057 Rostock, Germany; marc-andre.weber@med.uni-rostock.de

**Keywords:** smoldering multiple myeloma, multiple myeloma, SLiM-CRAB-criteria, MRI, MRI protocol, whole-body MRI, spinal MRI, focal lesion, staging, treatment indication

## Abstract

**Simple Summary:**

According to the current SLiM-CRAB-criteria, asymptomatic multiple myeloma patients who show >1 focal lesion in MRI are now upstaged to multiple myeloma with treatment indication. While the anatomic coverage of MRI protocols differs from spine over spine plus pelvis to whole-body between different institutions, the cutoff for the number of focal lesions which is currently used for the staging decision is not adapted according to the protocol. We found that usage of different MRI protocols leads to markedly different staging decisions according to current SLiM-CRAB-criteria. Adapting the cutoff for the number of focal lesions according to the MRI protocol enables to select comparable risk groups when using different MRI protocols. The combination of >3FL in spine and pelvis or >4FLs in the whole body came closest to select patients with an 80% probability to develop CRAB-criteria within 2 years, which was the original goal of the IMWG.

**Abstract:**

The purpose of this study was to assess how different MRI protocols (spinal vs. spinal plus pelvic vs. whole-body (wb)-MRI) affect staging in patients with smoldering multiple myeloma (SMM), according to the SLiM-CRAB-criterion ‘>1 focal lesion (FL) in MRI’. In this retrospective study, a baseline cohort of 147 SMM patients with wb-MRI at initial diagnosis was investigated, including prognostic data regarding development of CRAB-criteria. Fifty-two patients formed a follow-up cohort with a median of three wb-MRIs. The locations of all FLs were determined and it was calculated how staging decisions regarding the criterion ‘>1 FL in MRI’ would have been made if only a limited anatomic area (spine vs. spine plus pelvis) would have been covered by the MRI protocol. Furthermore, subgroups of patients selected by different cutoff-protocol-combinations were compared regarding their prognosis for development of CRAB-criteria. With an MRI protocol limited to spine/spine plus pelvis, only 28%/64% of patients who actually had >1 FL in wb-MRI would have been rated correctly as having ‘>1 FL in MRI’. Fifty-four percent/36% of patients with exactly 1 FL in spine/spine plus pelvis revealed >1 FL when the entire wb-MRI was analyzed. During follow-up, four more patients developed >1 FL in wb-MRI; both limited MRI protocols would have detected only one of these four patients as having >1 FL at the correct timepoint. Having >1 FL in spine/in spine plus pelvis/in the whole body was associated with a 43%/57%/49% probability of developing CRAB-criteria within 2 years. Patients with >3 FL in spine plus pelvis and patients with >4 FL in the whole body had an 80% probability to develop CRAB-criteria within 2 years. MRI protocols limited to the spine or to spine plus pelvis lead to substantial underdiagnoses of patients who actually have >1 FL in wb-MRI at baseline and during follow-up, which influences staging and treatment decisions according to the current SLiM-CRAB criteria. However, given the spatial distribution of FLs and the analysis on clinical course of patients indicates that the cutoff for the number of FLs should be adopted according to the MRI protocol when using MRI for staging in SMM.

## 1. Introduction

For the development into symptomatic multiple myeloma (MM), two precursor states have been defined: monoclonal gammopathy of undetermined significance (MGUS) and smoldering multiple myeloma (SMM), the latter being defined by higher values of m-protein and/or monoclonal plasma cells in the bone marrow and showing a higher risk of progression than MGUS [1,2,3]. Before an updated version of the IMWG criteria was published in 2014, these ‘asymptomatic’ myeloma patients were not considered to have indication for therapy before end-organ damage defined by the CRAB-criteria occurred [1]. However, within the last decade, it has been demonstrated that high-risk SMM patients benefit from early therapy, significantly prolonging the time to development of end organ damage [4,5].

With the goal to prevent end organ damage before it occurs and extend survival, the IMWG updated the definition of MM in 2014 and introduced new myeloma defining events to indicate therapy in an earlier stage of the disease [6]. The presence of more than 1 FL in MRI has been shown to be of prognostic value in MGUS [7] and SMM [8,9,10]. Consequently, this MRI-criterion became one of the three new myeloma defining events known as the SLiM-criteria, and patients with >1 FL in MRI now have formal indication for systemic therapy [6]. While all three studies on the prognostic impact of FLs in SMM used >1 FL as cutoff and consequently this cutoff was proposed in the SLiM-criteria, it has to be noted that different MRI protocols were used between these studies. One study reported on the risk of having >1 FL in the whole body [8], while the two other studies reported on the risk of having >1 FL in only the spine [9,10]. Given the large difference in anatomical coverage between these different MRI protocols, the question is raised whether these cutoff-protocol-combinations actually select comparable patient subgroups.

According to current recommendations, patients with SMM should undergo wb-MRI at initial diagnosis and then yearly for at least 5 years [11]. While wb-MRI is the primary recommendation by the IMWG [11,12], it is the consensus that wb-MRI is not available in many healthcare systems worldwide [11]. Wb-MRI offers a large anatomical coverage, but is time consuming with a duration of 30–45 min and, thus, expensive. Spinal MRI or spinal plus pelvic MRI have a reduced scan time of 10–15 min/15–25 min and hence have lower costs. Consequently, the IMWG recommended spinal MRI [6] or spinal plus pelvic MRI [11,12] as an alternative when wb-MRI is not available. However, it has not yet been assessed whether or in which amount different MRI protocols lead to different staging decisions when applying SLiM-CRAB-criteria.

Therefore, the primary goal of this study was to investigate how different MRI protocols affect staging and consequently indication for systemic therapy in patients with suspected SMM according to the new SLiM-CRAB-criteria, both at initial diagnosis and during follow-up. In addition, it was investigated which clinical or imaging factors point out patients who are understaged by a limited MRI protocol and therefore should undergo wb-MRI to receive correct staging according to SLiM-CRAB-criteria.

The second aim of the study was to compare whether the criteria ‘>1 FL in spine’ vs. ‘>1 FL in spine plus pelvis’ vs. ‘>1 FL in the whole body’ select groups of similar risk regarding development of CRAB-criteria. Furthermore, we aimed to investigate how the cutoffs for each specific protocol should be adapted to isolate a group of patients with an 80% probability to develop CRAB-criteria within 2 years, which was the original demand of the IMWG when updating the definition of multiple myeloma by defining the new biomarkers of malignancy [6].

## 2. Patients and Methods

### 2.1. Patients

Since MRI of SMM patients is being recommended both for initial and follow-up assessment for at least 5 years [11], this retrospective study investigated the influence of the MRI protocol on staging both in the baseline and in the follow-up setting. Patients with SMM according to the 2003 IMWG guidelines [1] and who had undergone wb-MRI were identified from earlier study cohorts on baseline [8]/follow-up MRI assessment [13] at our institution, and were combined in this analysis. Patients with earlier treatment due to localized plasma cell tumor plus evidence of systemic disease were excluded, as these patients are considered to have distinct progression risks. Furthermore, not all of these patients with local plasma cell tumors had received radiotherapy with a dose of more than 40 Gy, which has been shown to be the preferable dose in order to receive complete local remission by a recent study [14]. This resulted in a baseline-cohort of 147 patients with MRI at initial diagnosis, 52 of which received follow-up wb-MRI and are referred to as follow-up cohort. The latter underwent a median of three MRIs, with a median interval of 14 months. In total, the dataset consisted of 261 wb-MRI studies. This retrospective analysis of baseline and follow-up MR imaging was conducted in accordance with the declaration of Helsinki and approved by the ethical review board of the medical faculty of the University Heidelberg (S-247/2012 and S-511/2016).

### 2.2. Imaging, Clinical and Laboratory Examinations

All included wb-MRIs were performed on one of two identical 1.5 Tesla MRI systems (Magnetom Avanto, Siemens Healthineers, Erlangen, Germany). Details on imaging protocol and patient positioning have been published elsewhere [15,16,17] and used phased-array, body-matrix surface coils (Siemens Healthineers, Erlangen, Germany). The total duration of the imaging protocol was approximately 40 min. No contrast agent was applied.

During the complete observation period, all patients underwent clinical follow-up every 3–6 months in our outpatient clinic, and no treatment was administrated before progression due to CRAB-criteria [18] occurred. Details on clinical follow-up of baseline [8] and follow-up [13] cohort have been described before. The clinical course regarding development of CRAB-criteria [18] of all patients has been updated in 2020 and censored on 1 January 2015, as introduction of SLiM-CRAB-criteria at our institution from that timepoint enabled induction of therapy without fulfillment of CRAB-criteria. Serum m-protein, serum free light-chain ratio (SFLC-ratio) and plasma cell infiltration (PCI) of the bone marrow from biopsy at the posterior iliac crest at the initial MRI were used to investigate whether these factors are of use to identify patient subgroups who might be underdiagnosed by a limited MRI protocol.

### 2.3. Image Analysis

All wb-MRIs were read by two radiologists of which one was a board-certified senior radiologist in the clinical setting. For the purpose of this study, an additional study read was performed to determine the localizations of all FLs, categorizing FLs into 13 anatomical regions (skull, cervical spine, thoracic spine, lumbar spine, sacral bone/coccyx, shoulder girdle, humerus, pelvis, femur, tibia, extraosseous soft-tissue lesions, ribs, sternum). FLs were defined as circumscribed areas ≥5 mm in size being hypointense on T1w tse sequences and hyperintense on T2w STIR sequences, outside the typical locations for degenerative changes.

### 2.4. Data Analysis

The precise initial division into 13 anatomical regions allowed grouping of several regions for further analysis. In this study, the anatomical area of the spine relates to the axial skeleton with cervical spine, thoracic spine, lumbar spine and sacral bone/coccyx, as used before [19]. The area of spine plus pelvis additionally includes the hip bones.

To analyze the influence of the MRI protocol on correct diagnosis regarding presence of the SLiM-criterion ‘>1FL in MRI’, it was analyzed how decisions regarding the detection of ‘>1FL in MRI’ would have been made when only the respective anatomic area had been covered by the limited MRI protocol.

Patients who are not correctly rated with >1 FL in a limited MRI protocol might nevertheless eventually receive correct staging according to SLiM-criteria, when, in addition, another SLiM-criterion, either SFLC-ratio ≥ 100 or PCI ≥ 60%, is present. Therefore, a subgroup analysis focusing on patients in which other SLiM-criteria are absent needs to be performed, and it needs to be compared whether this subgroup shows a lower proportion of patients who would be underrated by the limited MRI protocols than the overall cohort. As the examinations investigated were performed before introduction of SLiM-CRAB criteria in 2014 [6], MRI, bone marrow biopsy and SFLC-ratio were not standard in clinical practice at that time. While our institution performed MRI [7,8,13] and bone marrow biopsies [20] in asymptomatic myeloma patients to investigate their prognostic implications, the SFLC-ratio was neither a clinical standard nor focus of research at our institution at that time and consequently, data is sparse. As information on SFLC-ratio was available for only 30 of 147 patients, it was not reasonable to perform a subgroup analysis in the 22 patients with SFLC-ratio < 100. However, information on plasma cell infiltration was present for 123 of 147 patients, and a subgroup analysis was performed on 118 patients with <60% plasma cell infiltration.

### 2.5. Statistical Analysis

Groups were compared with Fisher’s exact test for categorical parameters and with the Mann-Whitney test for continuous parameters. Primary endpoint was time to progression (TTP) defined as time from initial MRI to progression to MM due to development of CRAB-criteria [18]. Kaplan-Meier estimates and Cox regression were used to assess the impact of FLs on risk of progression. The 2-year progression rate (2YPR; referring to progression due to development of CRAB-criteria) was defined as the estimated proportion of patients with progression due to CRAB-criteria within 2 years after initial MRI. Time-dependent sensitivity and false-positive rate (FPR) for progression after 2 years accounting for censored data [21] was calculated. Harrell’s c-index was used to compare prognostic discrimination of different MRI protocols. *p*-values < 0.05 were considered statistically significant. Analyses were performed using R software (version 3.6, available at https://www.R-project.org/).

## 3. Results

### 3.1. Spatial Distribution of Focal Lesions in Whole Body MRI

**Figure 1:**
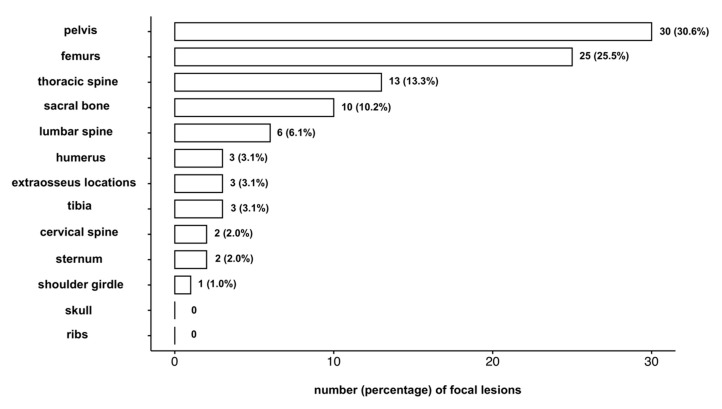
Spatial distribution of focal lesions in smoldering multiple myeloma patients. The number and percentage of focal lesions detected in each anatomical region is given, with overall 98 focal lesions detected in 36 of the 147 patients in the baseline cohort.

As a first step of this study, the spatial distribution of FLs on different anatomic regions of the body was analyzed. In the baseline-cohort of 147 patients, 111 patients did not show FLs, whereas 36 patients showed a total of 98 FLs. Thirty-one (31.6%) of these FLs were located in the spine, 61 (62.2%) were located in spine and pelvis. A detailed spatial distribution of FLs over the body at baseline is presented in Figure 1.

In the follow-up cohort, at the last MRI 33 patients were free of focal lesions, while 19 patients revealed a total of 54 FLs. Fifteen (27.8%) FLs were located in the axial skeleton, 30 (55.6%) were located in spine and pelvis.

### 3.2. Influence of MRI Protocol on Detection of More Than One Focal Lesion and Consequent Fulfillment of SLiM-Criteria

Results on detection of patients with >1 FL in wb-MRI in case that only a limited anatomic area is covered by the MRI protocol are demonstrated in Table 1.

In the baseline cohort, 25 of 147 patients revealed >1 FL in wb-MRI. Among these 25 patients, only seven patients showed >1 FL in the spine. This corresponds to a sensitivity of 28% for making the right diagnosis regarding presence of the MRI-SLIM-criterion ‘>1 FL in MRI’ when assessing only the spine instead of the whole-body, while 18 patients (72%) would have been understaged.

Among the 25 patients with >1 FL visible in wb-MRI, 16 would have been correctly recognized if the scan protocol had been limited to spine plus pelvis. This corresponds to a 64% sensitivity regarding presence of ‘>1 FL in MRI’, while nine patients (36%) of these 25 patients would have been understaged.

In the follow-up cohort, 10 patients already showed >1 FL at the initial wb-MRI. From the remaining 42 patients with either none or one single FL in their initial wb-MRI, four patients developed >1 FL in wb-MRI during imaging follow-up. Of these, only one would have been diagnosed correctly as having ‘>1 FL in MRI’ at the same timepoint during follow-up if MRI would have been limited to the spine, corresponding to a 25% sensitivity, while in the three remaining patients, the diagnosis as having ‘>1 FL in MRI’ would have been missed or delayed. If imaging had included the pelvis additionally, the results would have been the same.

In a subgroup analysis of the baseline cohort including only patients with PCI < 60%, the results were nearly identical (Table 2).

### 3.3. Can Clinical or Imaging Findings Reveal Patient Subgroups Who Are Not Diagnosed Correctly As Having >1 FL by a Limited MRI Protocol?

A subgroup analysis of the baseline cohort with division by number of detected FLs in the anatomic areas covered in the limited protocol is presented in Table 3. In patients where no FL was found in the spine, only 9% revealed >1 FL in wb-MRI. Of patients who had exactly 1 FL in the spine, more than half had >1 FL in the whole body. Patients who revealed one FL in the spine had a significantly higher risk to reveal >1 FL in the whole body compared to patients without FLs in the spine (*p* < 0.001). When no FL was apparent in either spinal or pelvic skeleton, only 4% of patients showed >1 FL in the whole body. If exactly 1 FL was detected in either spine or pelvis, more than one third of the respective patients had >1 FL in the whole body. Patients who revealed exactly one FL in spine plus pelvis had a significantly higher risk to reveal >1 FL in wb-MRI compared to patients without FLs in the spine (*p* = 0.003).

Furthermore, it was analyzed whether additional clinical parameters in patients who showed ≤1 FL in the limited imaging protocol were associated with showing >1FL in wb-MRI. In patients with ≤1 FL in the spine, the degree of bone marrow plasma cell infiltration was significantly higher in patients who revealed >1 FL in wb-MRI than in patients who did not (28.2% vs. 18.9%, *p* = 0.02). In patients with ≤1 FL in the spine plus pelvis, the degree of bone marrow plasma cell infiltration was also substantially higher in patients who revealed >1 FL in wb-MRI than in patients who did not, however, this did not reach statistical significance in this cohort (27.2% vs. 18.9%, *p* = 0.06). In patients with ≤1 FL in the limited imaging protocol serum m-protein levels and SFLC-ratio did not show significant differences between patients who revealed >1 FL in wb-MRI compared to those who revealed ≤1 FL in wb-MRI (all *p* > 0.05).

### 3.4. Risk for Development of CRAB Criteria in Patient Subgroups Selected by the Number of FLs in Different Anatomic Areas Covered by MRI

The clinical course regarding development of CRAB-criteria was investigated for patient subgroups that display a certain number of FLs in the respective anatomic area (Table 4). The subgroups which are selected when the cutoff ‘>1 FL’ is used in combination with different anatomic areas markedly differ regarding size, median time to progression and sensitivity to detect patients who develop CRAB-criteria within 2 years. The 2YPR of the subgroups selected by ‘>1 FL in spine’ vs. ‘>1 FL in spine plus pelvis’ vs. ‘>1 FL in the whole body’ are variable, but do not reach 80%. Only three out of 147 patients in this cohort showed more than two FL in the spine, and no adaption of the cutoff for the number of FLs in the spine was able to isolate a subgroup with approximately 80% 2YPR. However, >3 FL in spine plus pelvis or >4 FL in the whole body selected a subgroup with a 2YPR of 80%.

The prognostic discrimination of the number of FLs detected in the whole body (c-index = 0.63) is significantly higher than that of the number of FLs in the spine (c-index = 0.56, *p* = 0.01) and higher than that of the number of FLs in spine plus pelvis (c-index = 0.60, *p* = 0.08).

## 4. Discussion

This retrospective study on SMM patients demonstrates the essential influence of MRI-protocol on staging with the current SLiM-CRAB-criteria [6], with the severe consequence of missing or delaying induction of systemic therapy in a large proportion of patients when limited MRI protocols are used. Furthermore, an imaging finding from the limited MRI protocol was determined which identifies a subset of patients with a high risk of being understaged by the limited protocol. This enables to select these patients to undergo wb-MRI for correct staging according to the current SLiM-CRAB-criteria. Analyzing the clinical course for subgroups selected by different cutoff-protocol-combinations indicates that the cutoff should be adapted according to the respective MRI-protocol, to select comparable groups with 80% probability to develop CRAB-criteria within 2 years.

The role of MRI protocol in asymptomatic myeloma patients regarding fulfillment of SLIM-CRAB-criteria has not yet been investigated, and the information on the spatial distribution of FLs in asymptomatic myeloma is very limited in the literature. Bäuerle et al. have reported on the importance of wb-MRI in MM and MGUS. In the small subgroup of 6 MGUS patients with FLs, two patients had merely lesions in the spine, two had FLs only in the extraaxial skeleton and two had FLs in both locations [19]. Furthermore it has been reported that there are more patients showing merely extraaxial than merely axial FL in both MGUS [7] and SMM [8]. These earlier results pointed out the importance to image the extraaxial skeleton to correctly assess the actual tumor load and motivated the present study.

The presented data evaluating 261 wb-MRIs from 147 patients with SMM reveals that in both baseline and follow-up assessment the choice of MRI protocol leads to substantial differences regarding detection of patients with ‘>1 FL in MRI’, which under the current SLiM-CRAB-criteria is both stage- and therapy-deciding for these patients. Using spinal MRI, more than 70% of patients who already revealed >1 FL in wb-MRI are not rated correctly with ‘>1 FL in MRI’. Adding the pelvic skeleton to a spinal MRI protocol will, according to our findings, double the rate of correctly recognized patients with more than one FL. However, one third of all patients will remain understaged. In the follow-up setting, the influence of the MRI protocol on staging patients was also substantial, with limited MRI protocols underdiagnosing three out of four patients.

The question whether patients who are not correctly rated with >1 FL in a limited MRI protocol would finally still receive correct staging due to additional presence of another SLiM-criterion needs to be addressed. The subgroup analysis on 118 patients with <60% PCI revealed that the rate of underdiagnosis by a limited MRI protocol is identical in patients with <60% PCI compared to the overall cohort. This indicates that the understaging by limited MRI protocols is independent from the additional presence or absence of the SLIM-criterion >60% PCI. Due to sparse data on SFLC-ratio in this cohort, the question whether assessment of the SLIM-criterion ‘SFLC-ratio ≥100′ reduces the proportion of patients who get underdiagnosed by a limited MRI protocol will need to be answered by future studies.

The decision about the MRI protocol is a multidisciplinary task between requesting hematologist/oncologist, radiologist and patient, with additional influence from medical guideline commissions and financial aspects of the respective health system. Given the essential understaging when applying limited MRI protocols as demonstrated above, but also the limitations of financial budget and MRI scan time in all health systems worldwide, we face the clinical problem of how to effectively identify patients who are understaged by limited MRI protocols. Protocol extension in patients with one FL in the limited protocol is an efficient way to raise the proportion of correctly detected progressions regarding the current SLIM-CRAB-criteria with limited resources, as shown in the supplement in an exemplary cost-benefit modelling (Table S1). However, when applying the scheme of initially performing spinal/spinal plus pelvic MRI and extending the protocol in case of finding exactly one FL, still 44%/20% of patients would remain underdiagnosed according to our results, which points out that wb-MRI clearly remains the gold standard and should be performed if available. Additionally, when wb-MRI is not available but wb-CT is performed to exclude osteolysis at initial diagnosis, the CT does have some ability to detect focal bone marrow infiltrates in the appendicular skeleton [22], however the sensitivity of CT compared to MRI for these lesions is currently unclear, and their prognostic implication in SMM patients needs to be investigated.

Clinical course analysis confirmed that the application of >1 FL in combination with different MRI protocols results in selection of markedly different subgroups, especially regarding size, median time to development of CRAB-criteria and sensitivity to detect patients who develop CRAB-criteria within 2 years. Irrespective of the anatomic area included in the MRI-protocol, the 2YPR of patients selected by ‘>1 FL’ did not reach 80%, which was the original goal of the IMWG for biomarkers of malignancy [6]. No cutoff for the number of FLs in the spine was able to select patients with approximately 80% 2YPR, while patients with >3 FL in spine and pelvis or patients with >4 FL in the whole body revealed an 80% 2YPR. The hazard ratio of patients with >1 FL in the whole body is somewhat lower (2.96 vs. 4.04) compared to the earlier study from our institution [8]. This variation can be explained by exclusion of patients with a concomitant plasma cell tumor in comparison to the former study, and variations due to inclusion of additional patients receiving MRI between 2010 and 2013 at our institution and the updated follow-up data. The HR of >1 FL in the spine in this study is lower than the HR of >1 FL in the whole body, which is contrary to intuition, considering that patients with >1 FL in only the spine would be suspected to have additional FL elsewhere and thereby, on average, have a higher tumor load compared to the patient group with >1 FL in the whole body. Consequently, the subgroup ‘>1 FL in the spine’ would be suspected to have a higher risk of progression. With 1.93 (95% CI 0.60–6.22) the HR of seven patients with >1 FL in the spine in this study is also lower than in the two earlier studies investigating the prognostic value FLs in spinal MRI. Dhodapkar and colleagues reported on nine out of 156 patients with >1 FL in the spine demonstrating a HR of 4.71 (95% CI 1.57–14.11) [9]. Kastritis and colleagues reported on nine out of 67 patients with >1 FL in the spine having a HR of 7.2 (95% CI 3–18) [10]. Given the small number of patients in the high risk-groups and consequently the large 95% confidence intervals in all studies on MRI in SMM implies that selection of cutoffs on the limited available data is associated with uncertainty. However, cutoff selection remains necessary to translate findings on risk factors to clinical decision making. Especially considering only 28% of FL where located in the spine in this study, it seems intuitive to use different cutoffs when different MRI protocols are applied. Based on the cohort of this study, >3 FL in spine plus pelvis or >4 FL in the whole body came closest to the IMWG demand to select patients with approximately 80% probability to develop CRAB-criteria within 2 years. Furthermore, beyond definition of a specific cutoff for the number of FLs, this study revealed that wb-MRI has a significantly higher prognostic discrimination compared to spinal MRI.

The presented work has the following limitations: this was a retrospective study and the time between MRIs in the follow-up setting was variable. Therefore, our data did not allow quantification by how much time a limited protocol delayed the diagnosis of having >1 FL, as patients who reveal >1 FL in wb-MRI but have ≤1 FL in the limited MRI would presumably develop >1 FL in the limited MRI protocol over the course of time. Furthermore, sparse data on SFLC-ratio did not allow investigation of which proportion of patients underdiagnosed by a limited MRI protocol might be staged correctly, due to parallel showing a SFLC-ratio ≥100, which should be investigated by future studies. However, excluding patients showing the SLiM-criterion ≥60% PCI did not change the rate of underdiagnosed patients by a limited MRI protocol. As stated above, reporting hazard ratios and cutoff selection on limited cohort sizes has inherent uncertainty, which is represented by the 95%-CIs in this study.

## 5. Conclusions

MRI protocols limited to the spine or to spine plus pelvis lead to substantial underdiagnoses of patients who actually have >1FL in wb-MRI, with the severe consequence of missing or delaying onset of indicated therapy under the current SLiM-CRAB-criteria. Spinal plus pelvic imaging doubles the detection of patients with >1FL compared to spinal MRI only, but is substantially inferior to wb-MRI. Especially in patients with 1FL detected in a limited MRI protocol, the protocol should be extended to wb-MRI to perform correct staging according to the current SLiM-CRAB-criteria.

In the cohort of this study, the cutoff-protocol-combinations of either >3FL in spine plus pelvis or >4FL in the whole body both selected patients with 80% probability to develop CRAB-criteria within 2 years, which was the original goal of the IMWG when introducing the SLiM-criteria. While cutoff selection based on small study cohorts has inherent uncertainty, the distribution of FLs in SMM patients observed in this study and the analysis on clinical course of patient subgroups selected by different cutoff-protocol-combinations indicate that the cutoff for the number of FLs should be adapted according to the MRI protocol when using MRI for staging in SMM.

## Figures and Tables

**Table 1:** Performance of MRI protocols limited to spine / spine plus pelvis regarding detection of patients with >1 focal lesion (FL) in whole-body MRI.

Cohort	Anatomic Area Covered in Limited MRI Protocol	No. of Patients with >1FL in wb-MRI	No. of Patients with >1FL in Limited MRI Protocol	Sensitivity of Limited MRI Protocol ^1^
Baseline	spine	25	7	28%
	spine and pelvis	25	16	64%
Follow-up	spine	4	1	25%
	spine and pelvis	4	1	25%

^1^ regarding detection of patients with >1FL in wb-MRI.

**Table 2:** Performance of limited MRI protocols in the subgroup of 118 patients with <60% plasma cell infiltration.

Subgroup of Patients with <60% PCI	Anatomic Area Covered in Limited MRI Protocol	No. of Patients with >1 FL in wb-MRI	No. of Patients with >1 FL in Limited MRI Protocol	Sensitivity of Limited MRI Protocol ^1^
Baseline	spine	22	6	27%
	spine and pelvis	22	14	64%

^1^ regarding detection of patients with >1 FL in wb-MRI.

**Table 3:** Correlation between findings in the limited MRI protocol and presence of >1 focal lesion (FL) in wb-MRI.

Criterion in the Limited MRI Protocol	*n*	No. (%) of Patients with ≤1 FL in wb	No. (%) of Patients with >1 FL in wb	No. of Protocol Extension Needed Per One Additional Detected Progression ^1^
0 FL in the spine	127	116 (91%)	11 (9%)	12
1 FL in the spine	13	6 (46%)	7 (54%)	2
0 FL in the spine or pelvis	120	115 (96%)	5 (4%)	24
1 FL in the spine or pelvis	11	7 (64%)	4 (36%)	3

^1^ In the last column, the number of extensions from limited protocol to wb-MRI which would be needed to diagnose one additional progression (which would have been missed by the limited MRI protocol) for each subgroup is calculated.

**Table 4:** Risk for development of CRAB-criteria in patient subgroups selected by a specific number of FL in the respective anatomic area.

Anatomic Area	Spine	Spine Plus Pelvis	Whole-Body	Spine Plus Pelvis	Whole-Body
cutoff	>1 FL	>1 FL	>1 FL	>3 FL	>4 FL
*n* subgroup	7	16	25	5	5
Hazard ratio [95%-CI ^1^]	1.93 [0.60, 6.22]	2.83 [1.36, 5.87]	2.96 [1.61, 5.46]	4.50 [1.61, 12.58]	4.50 [1.61, 12.58]
*p*-value	0.264	0.004	<0.001	0.002	0.002
Sensitivity ^2^ (%)	9	29	38	13	13
FPR ^3^ (%)	3	6	11	1	1
2YPR ^4^ in (%) [95%-CI ^1^]	42.9 [0.0, 69.9]	57.1 [23.8, 75.9]	48.6 [24.4, 65.0]	80.0 [0.0, 96.5]	80.0 [0.0, 96.5]
median TTP ^5^ (months)	n.a.	15	34	8	8

^1^ CI: confidence interval. ^2^ Sensitivity to detect patients who develop CRAB-criteria within 2 years. ^3^ FPR: false positive rate. ^4^ 2YPR: 2-year progression rate (regarding development of CRAB-criteria). ^5^ TTP: time to progression (regarding development of CRAB-criteria). n.a.: not available: median time to progression was not reached in this subgroup.

## Supplementary Materials

The following are available online at https://www.mdpi.com/2072-6694/12/9/2537/s1, Table S1: Cost-benefit modelling for different MRI-protocol workflows.
