# Supplementary material for: Spatial Distribution of Focal Lesions in Whole-Body MRI and Influence of MRI Protocol on Staging in Patients with Smoldering Multiple Myeloma According to the New SLiM-CRAB-Criteria
Source: Cancers (Basel). 2020 Sep 7;12(9):2537. doi: 10.3390/cancers12092537 (PMC7563298; doi:10.3390/cancers12092537)
Supplement: Supplementary file 1 [file cancers-12-02537-s001.pdf]

cancers

Supplements

Spatial distribution of focal lesions in whole-body MRI and influence of MRI protocol on staging in patients with smoldering multiple myeloma according to the new SLiM-CRAB-Criteria

Markus Wennmann, Thomas Hielscher, Laurent Kintzelé, Bjoern H. Menze, Georg Langs, Maximilian Merz, Sandra Sauer, Hans-Ulrich Kauczor, Heinz-Peter Schlemmer, Stefan Delorme, Hartmut Goldschmidt, Niels Weinhold, Jens Hillengass, Marc-André Weber

Cost-benefit-modelling of different MRI protocol workflows in patients with SMM for diagnosis of progression to MM based on the criterion 'presence of >1FL in MRI'

Cost-benefit modelling for the application of different MRI protocol strategies is calculated. The models base on the spatial distribution of FLs observed in the presented baseline cohort of SMM patients. For modelling, assumptions about estimated MRI costs have to be made. Costs used in this model are based on the remuneration from German statutory and private health insurances for MRI protocols similar to the protocols used in our study. Moreover, remuneration for similar MRI examinations performed for study purposes in SMM / MM patients were taken into account. The authors further stress that costs of MRI can vary tremendously between countries and healthcare systems, thus limiting the external validity of the cost-benefit-modelling. The exemplary cost assumptions used in the presented modelling are:

- 300€ for spinal MRI
- 600€ for spinal and pelvic MRI
- 1100€ for a whole-body MRI (including sagittal spinal sequences)

Based on the assumptions made above, the average diagnostic cost per identified case of progression using each respective workflow is displayed in Table S1. Extending the protocol in cases where exactly 1FL is detected in the limited MRI protocol lead to markedly reduced cost per additionally diagnosed progression.

Table S1. Cost-benefit modelling for different MRI-protocol workflows.

**MRI protocol**	Percentage of Patients Correctly Diagnosed with >1FL [3]	Percentage of Patients with >1FL Who are Underdiagnosed[3]	Total Cost of Imaging Studies Based on Presented Study Cohort [€]	Average Diagnostic Cost per Identified Case of Progression due to >1FL in MRI [€]	Average Diagnostic Cost per Additionally Diagnosed Case of Progression[4] [€]
spinal MRI	28% (7/25)	72% (18/25)	44100	6300	/
spinal & pelvic MRI	64% (16/25)	36% (9/25)	88200	5513	/
wb-MRI	100% (25/25)	0	161700	6468	/
first spinal MRI → wb-MRI[1]	56% (14/25)	44% (9/25)	54500	3893	1486
first spinal & pelvic MRI → wb-MRI[2]	80% (20/25)	20% (5/25)	93700	4685	1375

[1] Diagnostic pathway: All patients undergo spinal MRI. In case a patient reveals exactly 1FL in the spine, the MRI protocol is extended to a whole-body MRI. [2] Diagnostic pathway: All patients undergo spinal and pelvic MRI. In case a patient reveals exactly 1FL in spine and pelvis, the MRI protocol is extended to a whole-body MRI. [3] The number of patients correctly diagnosed as having >1FL in MRI by the respective imaging protocol and the number of patients having >1FL in the whole body are given in parentheses. [4] in comparison to only performing the limited MRI protocol without extension to wb-MRI in cases where exactly 1FL is detected in the limited MRI protocol
